# Synthesis of poly(disulfide)s with narrow molecular weight distributions *via* lactone ring-opening polymerization†

**DOI:** 10.1039/d0sc00834f

**Published:** 2020-04-16

**Authors:** Sungwhan Kim, Kamila I. Wittek, Yan Lee

**Affiliations:** Department of Chemistry, College of Natural Sciences, Seoul National University Seoul 08826 Korea gacn@snu.ac.kr; Department of Chemistry, Johannes Gutenberg-University Mainz Duesbergweg 10-14 55128 Mainz Germany

## Abstract

We report the first example of controlled polymerization of poly(disulfide)s with narrow molecular weight distributions. 1,4,5-oxadithiepan-2-one (OTP), a disulfide-containing 7-membered ring lactone, was polymerized by using the diphenylphosphate (DPP) catalyzed lactone ring-opening polymerization method. The polymerization proceeded in a living manner, and the resulting polymers displayed very narrow polydispersity index (PDI) values below 1.1 and excellent backbone degradability responding to reducing conditions and UV irradiation.

## Abstract 2

We report the first example of controlled polymerization of poly(disulfide)s with narrow molecular weight distributions.

## Graphical abstract

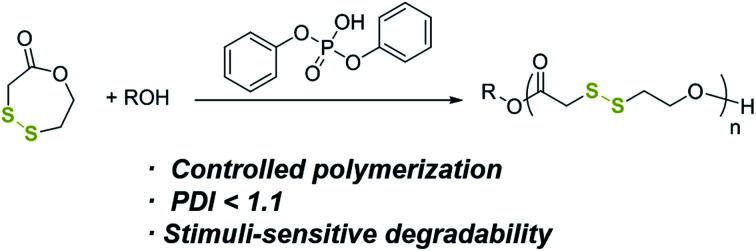

## Introduction

The disulfide bond is often regarded as a dynamic covalent bond with conditional reactivity or meta-stability.^1^ It can sufficiently maintain molecular integrity, being a reasonably strong bond with typical bond dissociation energies of 60 kcal mol^−1^. On the other hand, it can be easily cleaved and exchanged with other bonds in the presence of physical (*i.e*. light,^2^ heat,^3^ mechanical force,^4^*etc*.) or chemical (*i.e*. radicals,^5^ nucleophiles,^6^*etc.*) stimuli overcoming the dissociation energy barrier. In particular, the reversibility of oxidative coupling and reductive degradation between a disulfide and two thiols is a key factor in the structural integrity and controlled reactivity of proteins.^7,8^

The intriguing properties of disulfides have been popularly studied for application in the fields of polymer and materials science. Disulfide acts as a cross-linker between polymeric chains to improve the stiffness of polymeric networks, as shown in vulcanized rubber^9^ or disulfide-based hydrogels.^10^ Various pharmaceuticals can be conjugated to polymers or proteins *via* disulfide bonds for drug delivery.^11,12^ When appropriate signals are applied to the disulfide-based materials in a spatiotemporal manner, the disulfide bonds degrade either to disintegrate the polymer network structure or to release the active pharmaceuticals from the delivery carrier. UV-responsive self-healing gels,^13^ nanogels,^14^ and antibody–drug conjugates^12^ responding to high glutathione concentrations around cancerous tissues or in the cytosol are all on the frontier of materials and pharmaceutical sciences.

Notwithstanding the recent rapid progress in polymer science, the synthesis of fine polymers containing disulfide backbones is very limited, probably due to the vulnerability of disulfide bonds under most polymerization conditions. Oxidative coupling of disulfhydryl monomers,^15^ condensation polymerization of disulfide-pre-containing monomers^16^ and copolymerization of dihalide-containing monomers with sodium disulfide^17^ are representative strategies for the synthesis of poly(disulfide)s. However, the aforementioned step-growth polymerization methods inevitably lead to polymers only with a broad distribution of molecular weights (polydispersity index (PDI) > 2). Chain-growth polymerization has been attempted to obtain poly(disulfide)s with more narrow molecular weight distributions (MWDs), but only with limited improvement. For instance, ring-opening polymerization of strained disulfide monomers *via* thiol–disulfide exchange often produced mixtures of linear and cyclic polymers and even catenanes with broad MWDs.^18–22^ The formation of these complex product mixtures may be attributed to the limited selectivity of the thiol–disulfide exchange between disulfides in the monomer and the polymer backbone. Various reaction conditions were examined to overcome this problem and obtain linear polymers as a major product, but undesired cyclic oligomers were still present with PDI > 1.4.^22^ Ring-opening metathesis polymerization (ROMP), which is orthogonal with the thiol–disulfide exchange reaction, was also applied for the synthesis of poly(disulfide)s. However, undesirable interactions between the ruthenium catalyst and disulfides caused not only a large PDI > 1.5 but also a failure in homopolymerization of the disulfide monomer.^23^ From the previous results, we inferred that it is critical to choose polymerization conditions where unwanted thiol–disulfide exchange and catalyst–disulfide interactions are inhibited for obtaining fine poly(disulfide)s with narrow MWDs.

## Results and discussion

**Scheme 1:**
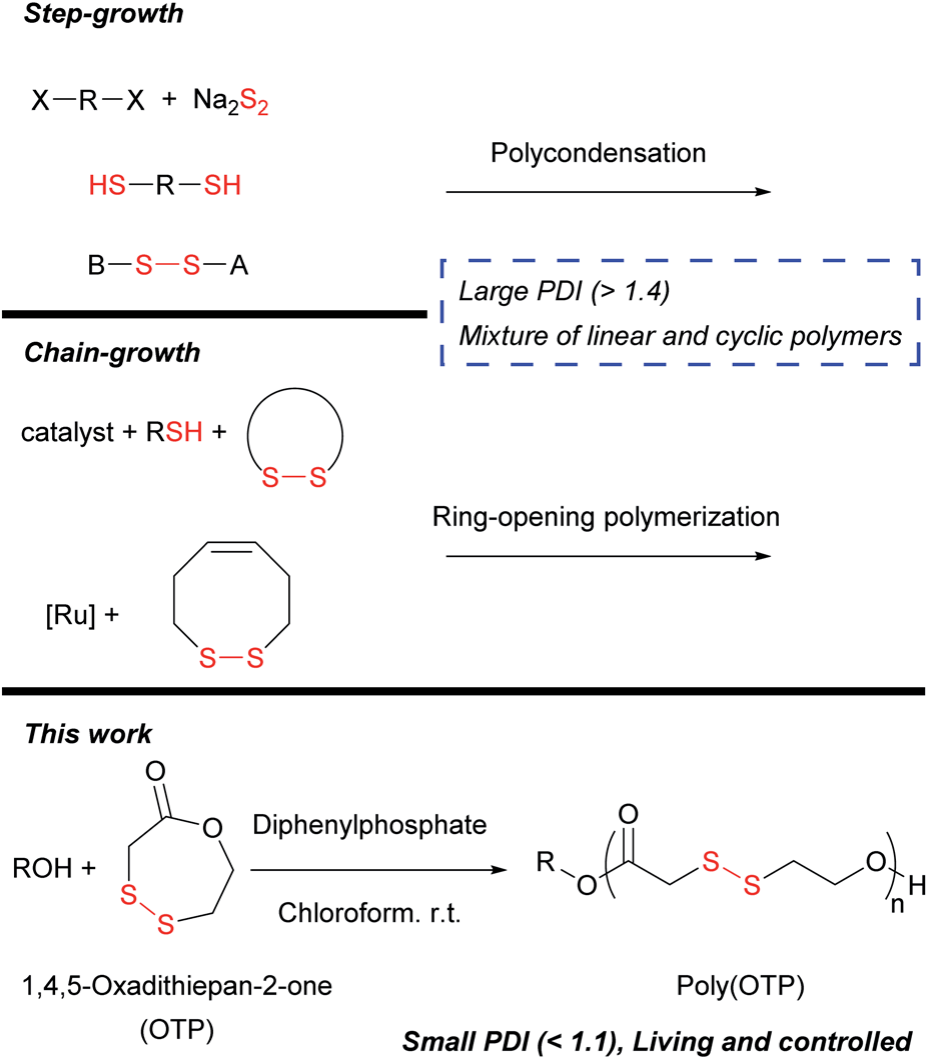
Polymerization of 1,4,5-oxadithiepan-2-one (OTP).

In this research work, we selected lactone polymerization for the synthesis of poly(disulfide)s since numerous catalysts for the controlled polymerization of poly(lactone)s have already been established.^24,25^ As for the monomer, we designed 1,4,5-oxadithiepan-2-one (OTP), an analogue of ε-caprolactone (εCL), where two of the five methylene groups are replaced with a disulfide. We reasoned that the 7-membered ring structure may provide sufficient ring strain for polymerization (Scheme 1). OTP was prepared from the catalytic intramolecular oxidation^26^ of an α,ω-disulfhydryl ester formed from 2-mercaptoethanol and 2-thioglycolic acid (see the ESI†). We have screened various catalysts ranging from bases to Lewis and Brønsted acids for the OTP polymerization, but the disulfide bonds were vulnerable under most of the reaction conditions, as the catalysts induced undesirable nucleophilic attack on disulfides by enhancing the electrophilicity of disulfides or nucleophilicity of nucleophiles. Among them, diphenylphosphate (DPP), a Brønsted acid with a p*K*_a_ of 3.88 in DMSO,^27^ showed remarkable tolerance to disulfides but strong catalytic activity for the polymerization of OTP. Therefore, we used DPP as the catalyst in all the polymerization reactions below.

The polymerization of OTP was carried out with catalytic DPP and benzyl alcohol (BnOH) as the initiator (I) at various [OTP]/[I] ratios (from [OTP]/[I] = 20 to 180). The general procedure for the polymerization is described in detail in the ESI.† We chose chloroform as the reaction solvent due to the solubility issue of the OTP polymer with a high sulfur content of approximately 43% (w/w). The MWDs were examined by size exclusion chromatography (SEC) (Table 1 and S1, ESI†). Surprisingly, the polymers (**P1–P5**) were found to have very narrow MWDs (PDI < 1.1) throughout the wide range of the monomer (M) to initiator ratio. The PDI values of poly(OTP)s are even smaller than those of poly(εCL) or poly(δ-valerolactone) in previous reports where the same catalyst was utilized.^25^*M*_n_ values from the SEC analysis were in reasonable agreement with the target *M*_n_. Additionally, we could confirm the preservation of a well-oriented head-to-tail backbone without disulfide exchange and fidelity of the terminal benzyl group in poly(OTP)s by MALDI-MS and ^1^H NMR study (Fig. S7 and S13–S17, ESI†).

**Table 1:** Polymerization of OTP at various monomer (M) to initiator (I) ratios and with various initiators

Entry	Initiator	Target [M]/[I]	Time	Conversion (%)^a^	*M*_n,target._ (kDa)	*M*_n,SEC_ (kDa)	PDI
**P1**	BnOH	20	18	>99	3.11	3.08^b^	1.07^b^
**P2**	BnOH	40	24	>99	6.12	5.71^b^	1.05^b^
**P3**	BnOH	80	48	>99	12.1	11.2^b^	1.05^b^
**P4**	BnOH	120	72	>99	18.1	17.0^b^	1.04^b^
**P5**	BnOH	180	96	99	21.7	22.6^b^	1.03^b^
**P6**	Propargyl alcohol	50	32	>99	7.57	7.82^b^	1.05^b^
**P7**	2-Propanol	50	32	>99	7.57	8.79^b^	1.05^b^
**P8**	*m*PEO-OH (1 kDa)	50	32	>99	8.51	9.22^c^	1.05^c^
**P9**	*m*PEO-OH (2 kDa)	80	48	>99	14.0	13.1^c^	1.04^c^

a Determined by ^1^H NMR spectra.

b Determined by size exclusion chromatography (SEC), with chloroform as the eluent, calibrated with polystyrene (PS) standards.

c Determined by SEC, with tetrahydrofuran as the eluent, calibrated with PS standards.

To widen the applicability of our method, we investigated various alcohols for initiation. Propargyl alcohol also produced poly(OTP) (**P6**) with desired molecular weights and a narrow PDI (Fig. S2, ESI†), which gives access to future end group functionalization *via* the alkyne–azide click reaction.^28^ Next, when we utilized 2-propanol as the initiator, we could still obtain a narrow MWD (**P7**, PDI = 1.05) (Fig. S3, ESI†) although secondary alcohols are generally recognized to be unsuitable for rapid initiation.^29^ We assumed that the initiation rate of OTP, even by secondary alcohols, is significantly faster than the propagation rate, thus leading to the low PDI. Furthermore, ω-methoxy-capped polyethylene oxide (mPEO) was also used as a macroinitiator for the synthesis of a block copolymer. The polymerization resulted in mPEO-*b*-poly(OTP) with narrow MWDs (**P8** and **P9**) (Fig. S4 and S5, ESI†). These well-defined amphiphilic block copolymers with narrow MWDs would form controllable polymeric nanostructures with disulfide-based responsiveness for future applications.

**Fig. 1:**
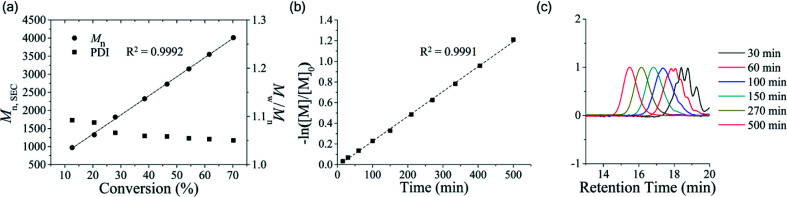
Controlled ring-opening polymerization of OTP. (a) *M*_n_ and PDI of poly(OTP) plotted against conversion. [OTP]/[BnOH] = 40, *M*_n_ and PDI were determined by SEC (chloroform as the eluent, PS standards). The conversion was determined by ^1^H NMR. (b) −ln([M]/[M]_0_) plotted against reaction time. [M]/[M]_0_ was determined by ^1^H NMR. (c) SEC chromatograms of poly(OTP)s changing over reaction time.

We then examined whether OTP is polymerized in a living/controlled manner. The polymerization kinetics of poly(OTP) were measured by ^1^H NMR and SEC (Fig. 1). We could observe a linear relationship between *M*_n_*versus* the conversion ratio and a decrease in the PDI (*M*_w_/*M*_n_) as the reaction progressed. Also, by plotting −ln([M]/[M]_0_) *versus* reaction time, we could obtain a strict linear relationship, which suggests that the polymerization rate is proportional to the OTP concentration in the first order.

**Fig. 2:**
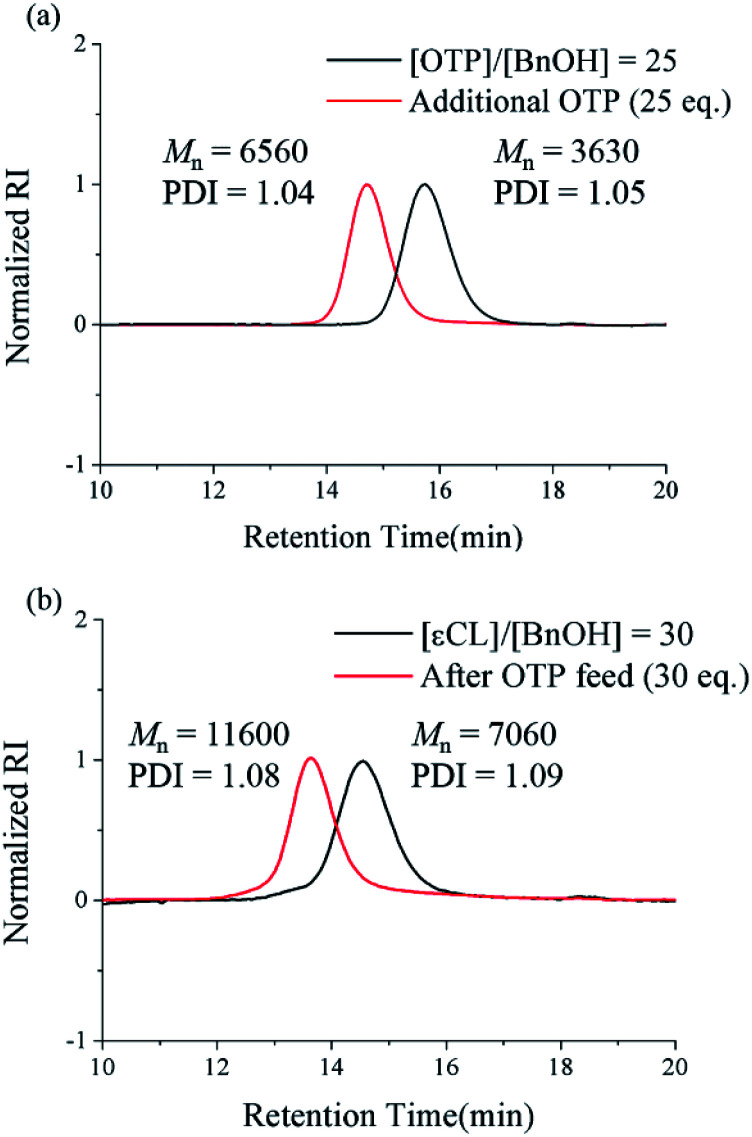
Successive one-pot post-polymerization of OTP. (a) The SEC chromatograms of poly(OTP)s prepared by one-pot post-homopolymerization. The first feed ([OTP]/[BnOH] = 25) and the second feed ([OTP]/[BnOH] = 25). (b) The SEC chromatograms of poly(εCL) and poly(εCL)-*b*-poly(OTP) prepared by one-pot post-copolymerization. The first feed ([εCL]/[BnOH] = 30) and the second feed ([OTP]/[BnOH] = 30). The black and red curves indicate the molecular weight distributions of polymers from the polymerization with only the first feed and with both the first and the second feeds, respectively.

We also performed one-pot post-polymerization with additional OTP feed to prove the livingness of the polymeric end group. First, we conducted polymerization at a [M]/[I] ratio of 25 for 24 h, where the full conversion of the monomer was confirmed by ^1^H NMR. The solution was further stirred without quenching for an additional 24 h. There was still no sign of backbiting or peak broadening in the SEC chromatogram even after 24 h from monomer depletion (Fig. 2a, black curve). Then, we added the second monomer solution feed ([M]/[I] = 25) to the unquenched polymer solution and stirred the mixture for an additional 48 h. We confirmed the second full consumption of the monomer by ^1^H NMR. The SEC chromatogram clearly showed the peak shift with an almost 2-fold increase of *M*_n_ from 3.63 kDa to 6.56 kDa, as well as the maintenance of the low PDI < 1.05 (Fig. 2a, red curve), which implies that the chain end of poly(OTP) is still “living”.

The results above encouraged us to attempt a successive ring-opening polymerization of εCL and OTP in a one-pot manner for the preparation of poly(εCL)-*b*-poly(OTP). After the first polymerization of the poly(εCL) block at a [εCL]/[I] ratio of 30 for 24 h, where the full conversion of εCL was confirmed by ^1^H NMR, the second OTP monomer was injected rapidly at an [OTP]/[I] ratio of 30. After the second polymerization proceeded for 48 h, we could observe that the SEC peak had shifted from the initial peak of poly(εCL) and that *M*_n_ showed an increase of 4.5 kDa (Fig. 2b). The PDI value was also maintained below 1.09 during the successive one-pot polymerization. In addition, the peak corresponding to the methylene proton at the chain end (–C***H***_2_OH) of poly(εCL) at 3.65 ppm (ref. 25) shifted to 4.15 ppm corresponding to those next to ester (–C***H***_2_OOC–) in the ^1^H NMR spectrum after the polymerization (see the ESI†), implying the successful one-pot post-polymerization with OTP from poly(εCL).

**Fig. 3:**
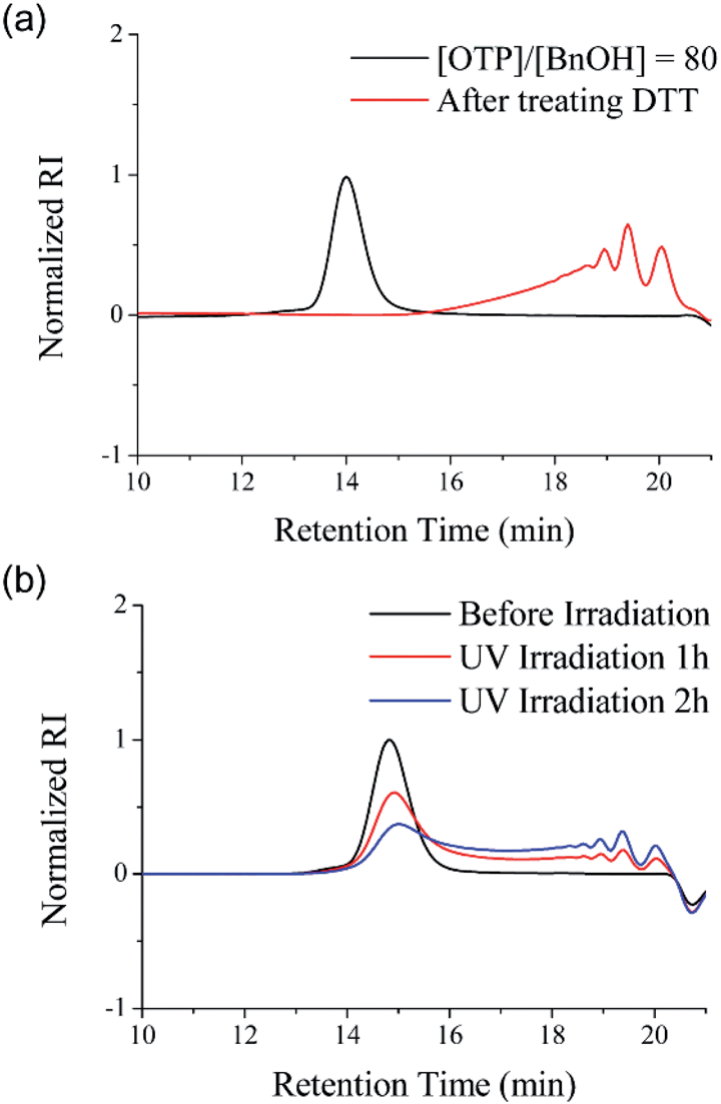
Stimuli-responsive degradability of poly(OTP)s. (a) The change of the SEC chromatogram of poly(OTP) (**P3**) before and after treatment with DTT (1 eq. of the disulfide bonds in **P3**). (b) The change of the SEC chromatogram of poly(OTP) (**P2**) before and after UV irradiation (5 W cm^−2^). The black curves indicate the molecular weight distributions of poly(OTP)s before stimuli-triggering.

The disulfide backbone of poly(OTP) is expected to have degradability responding to various chemical and physical stimuli. As a representative chemical stimulus to trigger the disulfide metathesis in poly(OTP), we chose D,L-dithiothreitol (DTT), a reagent well known to induce thiol–disulfide exchange reactions by forming a stable intramolecular cyclic disulfide while reducing other disulfide substrates.^30^**P3** (*M*_n_ = 11.2 kDa) was dissolved in chloroform and DTT was added to the solution at a molar ratio of 1 : 1 relative to the disulfide content of **P3**. After incubation at ambient temperature for 24 h, the backbone of poly(OTP) was almost completely degraded to oligomers, as shown in the SEC chromatogram in Fig. 3a. Poly(OTP) showed the characteristic degradability of disulfide polymers responding to the reducing conditions. Then, we also examined the backbone degradation of poly(OTP) by UV irradiation, which is known to facilitate disulfide metathesis by generating thiyl radicals *via* the homolytic cleavage of disulfide.^2^ After irradiation with UV light (*λ*_max_ = 357 nm, 5 W cm^−2^), the initial peak of **P2** (*M*_n_ = 5.71 kDa) in the SEC chromatogram gradually decreased in intensity with an increase in retention time, while small peaks appeared with increasing intensity in the oligomer region (Fig. 3b). The results clearly exhibit the UV-responsive degradability of the poly(OTP) backbone. In addition, the disulfide bonds in the poly(OTP) backbone are quite stable even at 100 °C, where the broadening of the MWD was observed probably due to transesterification, but start to show exchange reactions to produce a mixture of head-to-head, head-to-tail and tail-to-tail disulfide bonds with larger broadening of the MWD at 120 °C (Fig. S11 and S12, ESI†).

## Conclusions

In summary, we have demonstrated the first example of controlled living polymerization of disulfide-backbone polymers with narrow MWDs (PDI < 1.1). We could successfully synthesize poly(OTP)s from various alcohol initiators and their block copolymers with PEO and poly(εCL) through the combination of a newly designed 7-membered disulfide-containing lactone, OTP, and a lactone-activating catalyst unreactive to disulfides. The poly(OTP)s showed characteristic degradability of disulfide-bearing compounds under exposure to thiols and UV irradiation. Thanks to the simple introduction of a poly(disulfide) backbone with a controlled length, the OTP polymerization can be a very useful tool for the development of smart materials with stimuli-responsive reversible degradability, especially in the biomedical fields. More importantly, we believe that poly(OTP)s can be applicable to supramolecular chemistry for formation of delicate nanostructures requiring narrow MWDs of the components, where the application of poly(disulfide)s with attractive stimuli-responsiveness has been difficult due to broad MWDs until now.

## Conflicts of interest

There are no conflicts to declare.

## Supplementary Material

SC-011-D0SC00834F-s001
