# Supplementary material for: Synthesis of poly(disulfide)s with narrow molecular weight distributions via lactone ring-opening polymerization
Source: Chem Sci. 2020 Apr 16;11(19):4882–6. doi: 10.1039/d0sc00834f (PMC8159261; doi:10.1039/d0sc00834f)
Supplement: SC-011-D0SC00834F-s001 [file SC-011-D0SC00834F-s001.pdf]

Electronic Supplementary Material (ESI) for Chemical Science.
This journal is © The Royal Society of Chemistry 2020

Electronic Supplementary Information(ESI) for

# Synthesis of Poly(disulfide)s with Narrow Molecular Weight Distributions via Lactone Ring-Opening Polymerization

Sungwhan Kim, Kamila I. Wittek and Yan Lee*

**Table of Contents**


**1. General** ........ **S2**

**2. Experimental procedures** ........ **S3—S6**

A. Synthesis of 1,4,5-oxadithiepan-2-one (OTP) ........ S3

B. General procedure for homopolymerization (**P1**-**P7**) ........ S4

C. General procedure for block-copolymerization using mPEO as a macroinitiator (**P8**, **P9**) ........ S5

D. Procedure for synthesis of Poly(εCL)-*b*-Poly(OTP) (Target BnOH : εCL : OTP = 1: 30 : 30) ........ S6

E. Procedure for degradation test of poly(OTP) with D,L-Dithiothreitol(DTT) ........ S6

F. Procedure for degradation test of poly(OTP) under UV irradiation ........ S6

**3. Supporting figures** ........ **S7—S12**

A. SEC chromatograms of polymers ........ S7—S9

B. MALDI-MS spectra of OTP and **P2** ........ S10

C. Time dependent $^1$H NMR spectra during the kinetics test ........ S11

D. Thermogravimetric analysis(TGA) of homopolymer **P5** ........ S12

**4. Supporting information for degradation experiments** ........ **S13—S18**

A. $^1$H NMR spectra after treating DTT ........ S13

B. Thermal broadening of molecular weight distribution upon heating ........ S14

C. Structural study for thermal broadening of molecular weight distribution ........ S15—S18

**4. NMR spectra of compounds** ........ **S19—S24**

**5. References** ........ **S25**

## 1. General

$^{1}H$-NMR spectra were recorded on a Varian (500 MHz) spectrometer. $^{1}H$ chemical shifts were referenced from the chemical shift of tetramethylsilane (0.00 ppm). $^{13}C$-NMR spectra were recorded on a Varian (125 MHz) spectrometer with complete proton decoupling. $^{13}C$ chemical shifts were referenced from the chemical shift of $CDCl_3$ (77.16 ppm). Two-dimensional (2D) NMR spectra of monomer and polymer were recorded on a Varian (500 MHz) spectrometer. 2D $^{1}H$-$^{1}H$ homonuclear and $^{1}H$-$^{13}C$ heteronuclear experiments [2D correlated spectroscopy (COSY) and heteronuclear single quantum coherence (HSQC)] were performed to assign the NMR spectra. MALDI-MS spectra were recorded on a Bruker Daltonics Microflex LT and a Bruker Daltonics UltrafleXtreme TOF/TOF using dithranol as a matrix and sodium trifluoroacetate as a cation source. UV light was generated from an Inno-cure 150, attached with a 357 nm-filter. ATR-FTIR spectra were recorded on a Thermo Scientific NICOLET iS10 spectrometer. For a long-time reagent infusion, LSP02-1B syringe pump was used. Size exclusion chromatography (SEC) was performed on a Young Lin YL9100 GPC System equipped with Shodex GPC columns [K-803 (for chloroform) or KF-803 (for tetrahydrofuran)]. Samples were diluted in 1-5 mg/ml by the mobile phase and filtered through a 0.20 µm-PTFE filter before injection into the SEC system. Thermogravimetric analysis (TGA) was carried out under $N_2$ gas at a scan rate of 10 °C/min with a Q50 model device from TA Instruments. 2-Mercaptoethanol, 2-thioglycolic acid, p-toluenesulfonic acid monohydrate (*p*-TsOH), diphenylphosphate (DPP), ε-caprolactone (εCL), benzyl alcohol (BnOH), propargyl alcohol, 2-propanol, poly(ethylene oxide) methyl ether ($M_n$ ~2000), D,L-dithiothreitol (DTT), dithranol, chloroform, sodium iodide and 4Å molecular sieves were purchased from Sigma Aldrich (USA) and poly(ethylene oxide) methyl ether ($M_n$ ~1000) was purchased from TCI (Japan). 1,3,5-trimethoxybenzene was purchased from Alfa Aesar (USA) Pyridine, magnesium sulfate, sodium thiosulfate, hydrogen peroxide (30.0 ~35.5 % in water), ethyl acetate, methanol, dichloromethane, diethylether, chloroform (HPLC grade), tetrahydrofuran (HPLC grade) was purchased from Samchun Chemicals (Korea). Alcohols and ε-caprolactone were purchased as an anhydrous form or distilled under calcium hydride before use.

## 2. Experimental Procedures

### A. Synthesis of 1,4,5-oxadithiepan-2-one (OTP)

1,4,5-oxadithiepan-2-one (OTP) was synthesized through two steps.

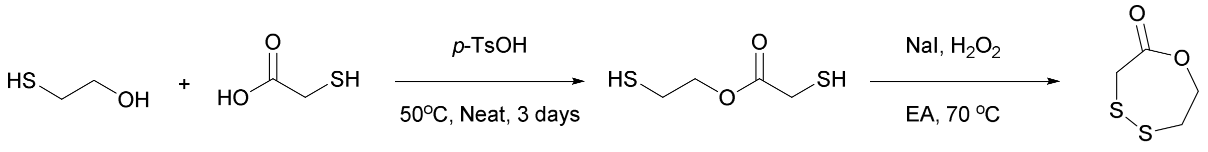


**Step 1.** Synthesis of 2-mercaptoethyl 2-mercaptoacetate

To 2-mercaptoethanol (1.00 ml, 14.2 mmol), 1.19 ml of 2-thioglycolic acid (17.0 mmol, 1.20 equiv.) and 135 mg of *p*-toluenesulfonic acid (0.710 mmol, 0.05 equiv.) was added. The reaction mixture was sealed and stirred at 50°C for 3 days. The crude mixture was dissolved in 20 ml of chloroform and washed with 100 ml of aq. sodium bicarbonate (distilled water : saturated $NaHCO_3$ in water = 9 :1) three times and then, with 100 ml of distilled water three times. Resulting organic layer was dried over anhydrous $MgSO_4$, filtered and concentrated under reduced pressure. After that, 10 ml of dichloromethane was added and concentrated under reduced pressure to evaporate residual chloroform. The product was pale yellow oil and used without further purification. (1.40 g, 65 %) $^1$H NMR (500Mz, $CDCl_3$) δ 4.27(t, 2H), 3.29(d, 2H) 2.78 (t, 2H) 2.03(t, 1H) 1.53(t, 1H)

**Step 2.** Catalytic oxidative cyclization of 2-mercaptoethyl 2-mercaptoacetate[1]

To 250 ml of ethyl acetate, sodium iodide (1.87 mg, 12.5 μmol) and 0.25 ml of hydrogen peroxide solution (30.0 ~35.5 % in water) was added. The mixture was stirred vigorously until the color of the solution changed to yellow. 190 mg of the product from step 1 was dissolved in 25 ml of ethyl acetate and infused to the sodium iodide mixture with syringe pump for 10 h at 70°C. After full infusion of the solution, the reaction mixture was cooled into room temperature and washed with 200 ml of 10 % aq. sodium thiosulfate once and with 250 ml of distilled water twice. The organic layer was dried over anhydrous $MgSO_4$, filtered and concentrated under reduced pressure. The resulting crude oil was purified by flash column chromatography on silica gel (ethyl acetate : *n*-hexane = 6 : 1) to afford 1,4,5-oxadithiepan-2-one as a pale yellow oil (114 mg, 60 %). $^1$H NMR (500Mz, $CDCl_3$) δ 4.66 (t, 2H), 3.93 (s, 2H), 3.09 (br, 2H). $^{13}$C NMR (125Mz, $CDCl_3$) δ 169.11, 69.25, 41.99, 36.75. FTIR (ATR) 3448, 2957, 1729, 1303, 1240, 1099, 1061, 1013, 774, 630, 561, 496, 454, 410 $cm^{-1}$ MS (MALDI-MS) calculated for $C_4H_6$ Na $O_2S_2$ $[M+Na]^+$: 173.20, found: 173.20. elemental analysis found: C, 32.22; H, 4.11; S, 43.66; O, 21.16. Calc. for $C_4H_6S_2O_2$: C, 31.98; H, 4.03; S, 42.69; O, 21.30 %

## B. General procedure for homopolymerization of OTP (P1-P7)

OTP (300 mg, 2 mmol) was dissolved in 1.7 ml of anhydrous chloroform in a glove box. 4Å molecular sieves were added to the solution and stored for 2 days under 15-17 $^{o}$C with argon atmosphere. Concentration of the monomer solution was calibrated by $^{1}$H NMR spectroscopy using 1,3,5-trimethoxybenzene as an internal standard. Diphenyl phosphate (DPP) and various initiators (alcohols) were separately dissolved into anhydrous chloroform to prepare 0.1 M stock solutions. 0.1 ml of the initiator stock solution was added to the solution with desired amount of OTP according for the target [M]/[I] ratio. 0.1 ml of the DPP stock solution was injected rapidly after the reaction mixture was stirred for 2 min. Reaction progress of the polymerization was determined with small aliquots taken from the reaction mixture. Aliquots were quenched immediately with excess amount of pyridine. Reaction mixtures were also quenched with excess amount of pyridine (> 5 equiv.) and precipitated into cold methanol. The precipitates were collected by centrifugation at 2500 rpm and dried *in vacuo* to afford white greasy solid regardless of the initial [M]/[I]. The dried polymers were stored at -20 $^{o}$C.

## C. General procedure for block-copolymerization using mPEO as a macroinitiator (P8 and P9)

Solutions of OTP and DPP were prepared by the same method in the homopolymerization. mPEO was dissolved in anhydrous chloroform and dried with 4Å molecular sieves for 3 days. The mPEO solution is calibrated by $^{1}$H NMR spectroscopy using 1,3,5-trimethoxybenzene as an internal standard and used as the stock solution. 0.01 mmol of mPEO in chloroform is added to the monomer solution and the mixture was stirred for 2 min. After that, 0.1 ml of the DPP solution was injected rapidly. Reaction was quenched with the same method in the homopolymerization. Polymer was precipitated into cold diethyl ether. The precipitates were collected by centrifugation at 2500 rpm and dried *in vacuo* to afford white greasy solid. The dried polymers were stored at -20 $^{o}$C.

$^{1}$H, $^{13}$C NMR Characterization of representative polymers from different initiators.

**P2** , I = BnOH, [OTP]/[I] = 40 in $^{1}$H NMR analysis

$^{1}$H NMR (500Mz, $CDCl_3$) δ 7.42-7.32 (m, 5H), 5.19 (s, 2H), 4.44 (t, 76H), 4.37 (t, 2H), 3.89 (t, 2H), 3.55(s, 76H), 3.53 (s, 2H), 3.51 (t, 2H), 3.04 (t, 76H), 2.96-2.94 (m, 4H).

**P6** , I = Propargyl alcohol, [OTP]/[I] = 51 $^{1}$H NMR analysis

$^{1}$H NMR (500Mz, $CDCl_3$) δ 4.77(d, 2H), 4.44 (t, 104H), 3.89 (t, 2H), 3.55(s, 104H), 3.53(s, 2H), 3.04 (t, 104H), 2.96 (t, 2H), 2.55(t, 1H).

**P7**, I = 2-Propanol, [OTP]/[I] = 55 in $^{1}$H NMR analysis

$^{1}$H NMR (500Mz, $CDCl_3$) δ 5.06(m, 1H), 4.44 (t, 108H), 3.89 (t, 2H), 3.55(s, 108H), 3.53(s, 2H), 3.04 (t, 108H), 2.96 (t, 2H), 1.28(d, 6H).

**P8,** I = mPEO-OH(1 kDa), [OTP]/[I] = 49.5 in $^{1}$H NMR analysis

$^{1}$H NMR (500Mz, $CDCl_3$) δ 4.44 (t, 97H), 4.31(t, 2H), 3.89 (t, 2H), 3.73 (t, 2H), 3.64 (s, 86H), 3.55(s, 97H), 3.53(s, 2H), 3.38(s, 3H), 3.04 (t, 97H), 2.96 (t, 2H).

$^{13}$C NMR Peaks from polymer backbone

$^{13}$C NMR (125Mz, $CDCl_3$) δ 169.23, 63.31, 41.45, 36.70.

FTIR and elemental analysis of **P2**

FTIR (ATR) 3450, 2949, 1728, 1264, 1143, 1120, 990, 767, 696, 576, 466, 435, 404 cm$^{-1}$

elemental analysis found: C, 32.04; H, 4.05; S, 42.13; O 20.94.Calc. for $C_{167}H_{247}S_{80}O_{81}$: C, 32.80; H, 4.07; S, 41.94; O ,21.19 %

## D. Procedure for synthesis of poly(εCL)-*b*-poly(OTP) (Target BnOH : εCL : OTP = 1: 30 : 30)

Solutions of OTP, DPP and BnOH were prepared with the same method in the homopolymerization. εCL was prepared as 1 M stock solution in chloroform. To 0.3 ml of the εCL solution, 0.1 ml of the BnOH solution was added. The mixture was stirred for 2 min and rapidly injected with 0.1 ml of the DPP solution. After confirming the full conversion of εCL with $^1$H NMR spectroscopy, 30 equiv.(to BnOH) the OTP solution was rapidly injected to the mixture. The reaction was quenched with the same method in the homopolymerization. Polymer was precipitated into cold methanol. The precipitates were collected by centrifugation at 2500 rpm and dried *in vacuo* to afford white greasy solid. The dried polymer is stored at -20 $^o$C.

$^1$H NMR Characterization of polymer

I = BnOH, [BnOH] : [εCL] : [OTP] = 1 : 33.5 : 35 in $^1$H NMR analysis

$^1$H NMR (500Mz, $CDCl_3$) δ 7.42-7.32 (m, 5H), 5.19 (s, 2H), 4.44 (t, 68H), 4.15 (t, 2H), 4.06 (t, 65H) 3.89 (t, 2H), 3.55 (s, 68H), 3.53 (s, 2H), 3.04 (t, 68H), 2.96 (t, 2H), 2.37 (t, 2H), 2.31 (t, 65H), 1.65 (m, 134H) 1.38 (m, 67H)

## E. Degradation test of poly(OTP) with D,L-Dithiothreitol(DTT)

To a solution of **P3** (7.5 mg) in chloroform (1 ml, HPLC grade) 7.7 mg of DTT was added. The solution was stirred for 24 h at room temperature and analyzed with SEC without further purification.

## F. Degradation test of poly(OTP) under UV irradiation

7.5 mg of **P4** was dissolved in chloroform (1 ml, HPLC grade). The solution of **P4** was irradiated by UV light ($\lambda_{max}$ = 357 nm, 5 W $cm^{-1}$) in open condition. The solution was analyzed with SEC at each timespots without further purification.

## 3. Supporting Figures

### A. SEC chromatograms of polymers

Column condition of SEC chromatogram in **Figure S1**, **S2**, and **S3**

: Shodex K-803 column, 0.5 ml/min chloroform, 50 °C and polystyrene as standard

Column condition of SEC chromatogram in **Figure S4**

: Shodex KF-803 column, 0.7 ml/min tetrahydrofuran, 50 °C and polystyrene as standard

Column condition of SEC chromatogram in **Figure S5**

: Shodex KF-803 column, 0.5 ml/min tetrahydrofuran, 50 °C and polystyrene as standard

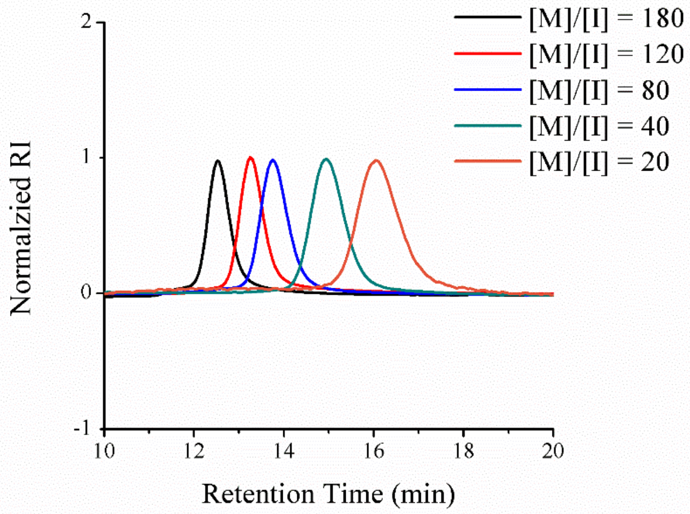


**Figure S1.** SEC chromatograms of **P1-P5**

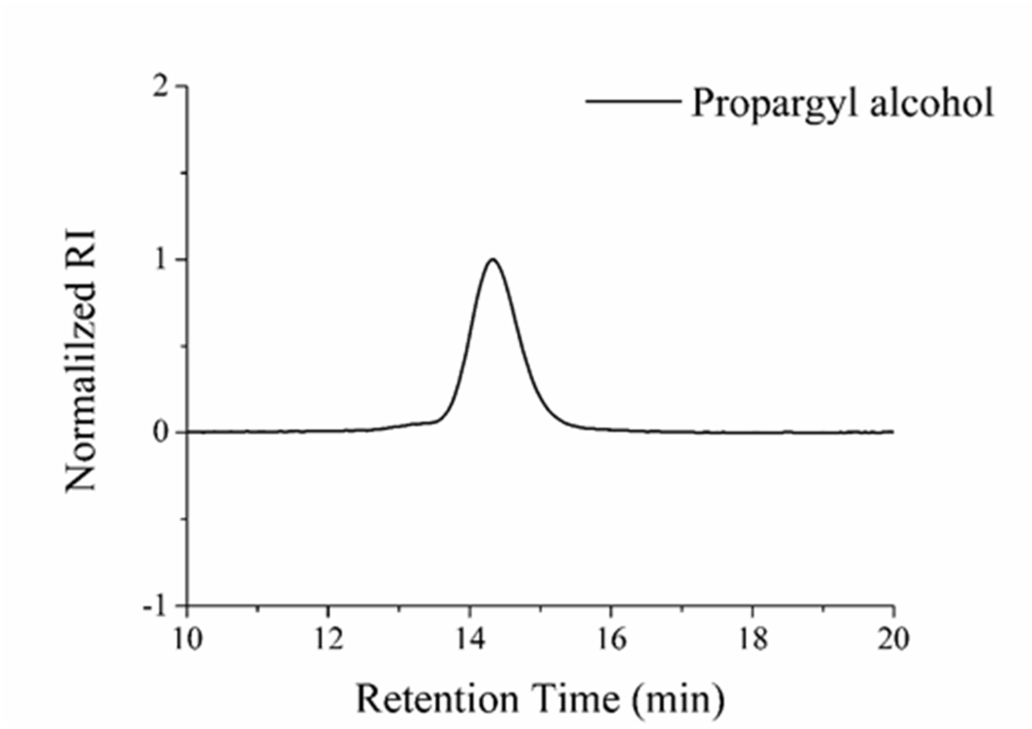


**Figure S2.** SEC chromatogram of **P6**

Target [M]/[I] = 50

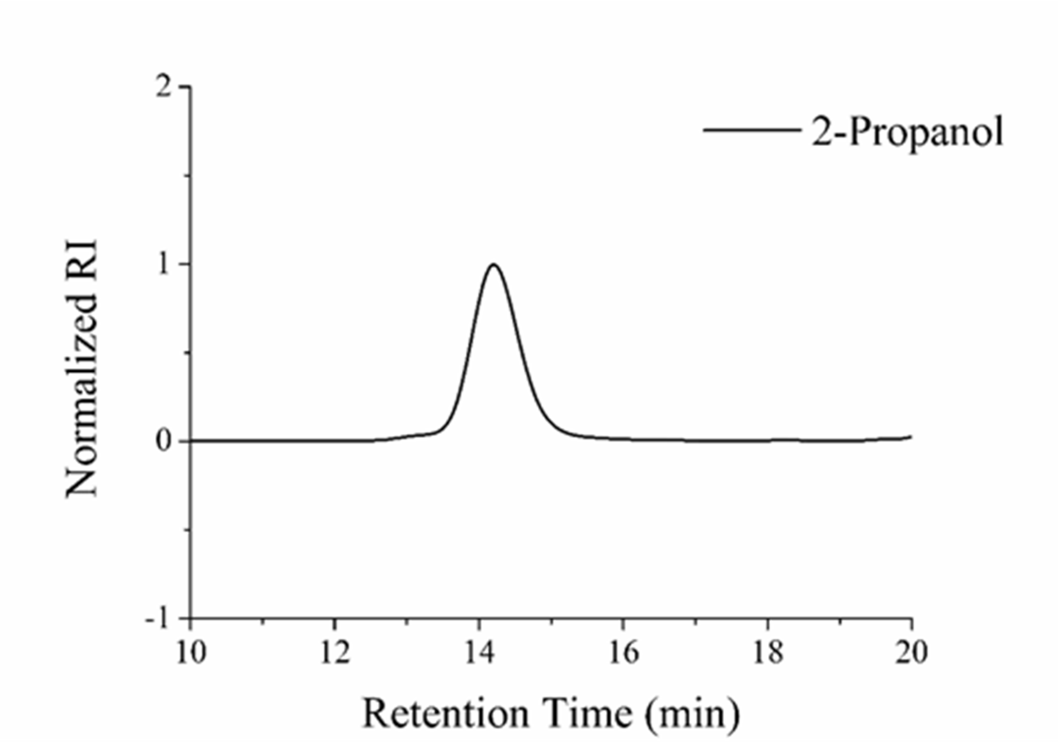


**Figure S3.** SEC chromatogram of **P7**

Target [M]/[I] = 50

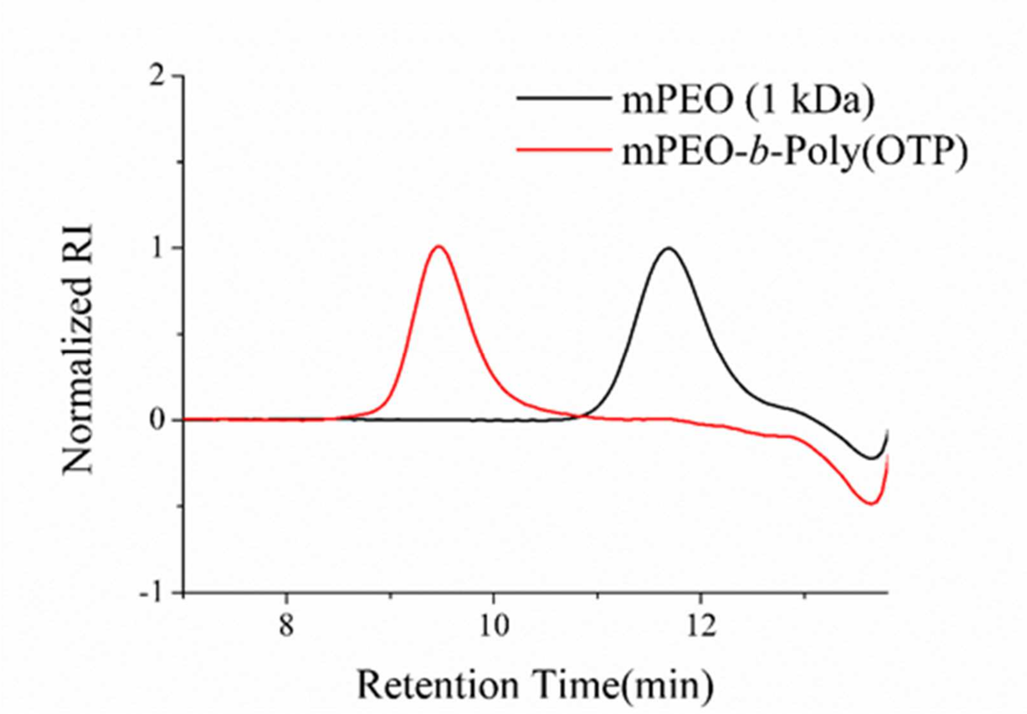


**Figure S4.** SEC chromatograms of **P8**

Target [M]/[I] = 50

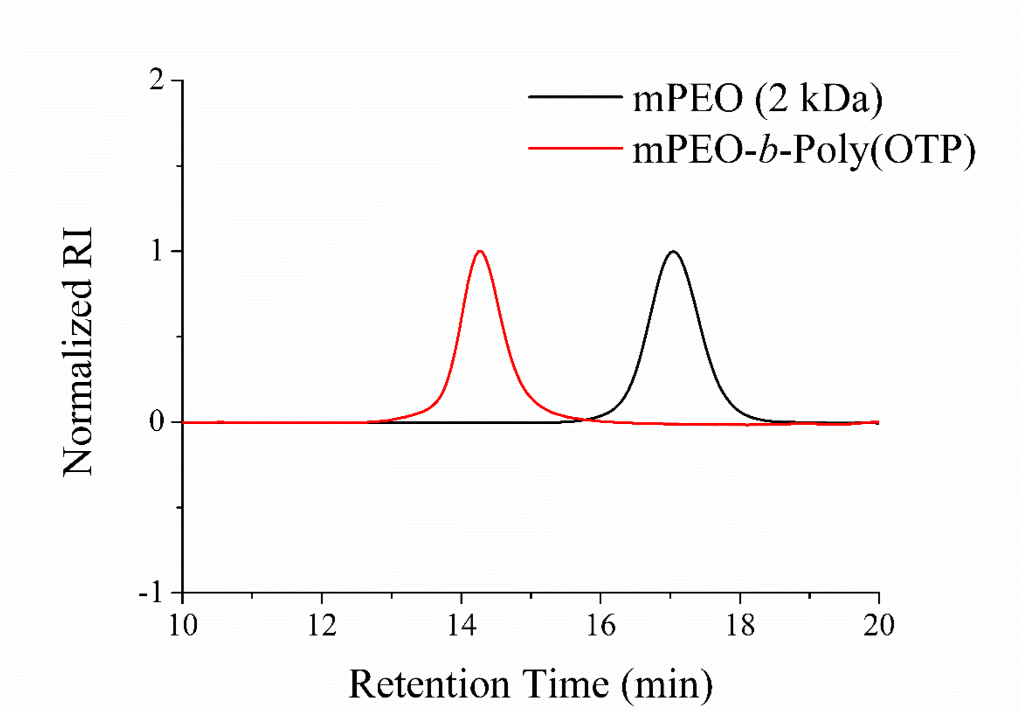


**Figure S5.** SEC chromatograms of **P9**

Target [M]/[I] = 80

## B. MALDI-MS spectra of OTP and P2

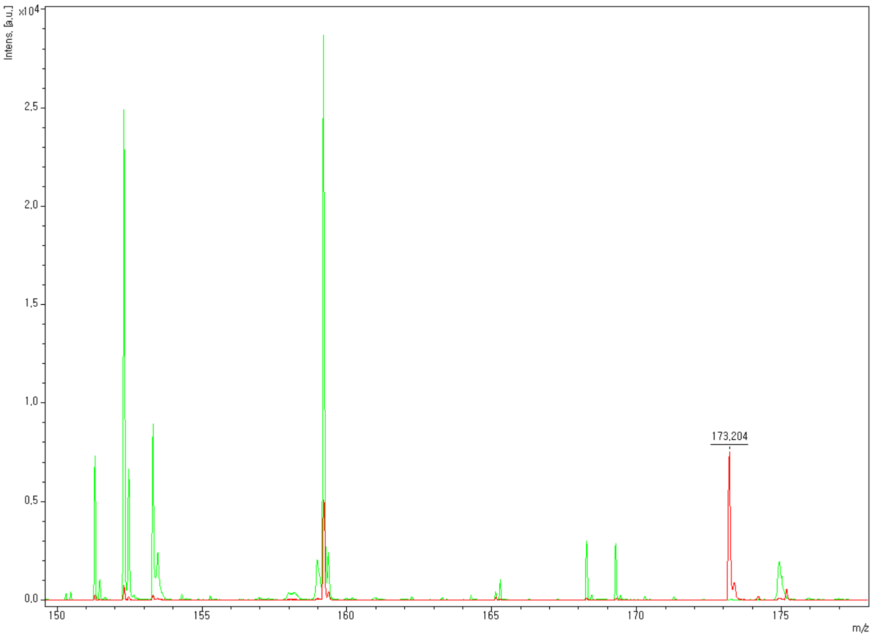


**Figure S6.** MALDI-MS analysis of OTP

Green: Peaks from matrix only (dithranol/sodium trifluoroacetate)

Red: Peaks from OTP with matrix

Calculated for $C_4H_6$ Na $O_2S_2$ $[M+Na]^+$: 173.20, found: 173.20

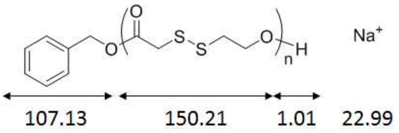


| | Theoretical value | Detected |
|---|---|---|
| 1 | 4787.64 | 4786.85 |
| 2 | 4937.85 | 4937.95 |
| 3 | 5088.06 | 5088.00 |
| 4 | 5238.29 | 5238.67 |

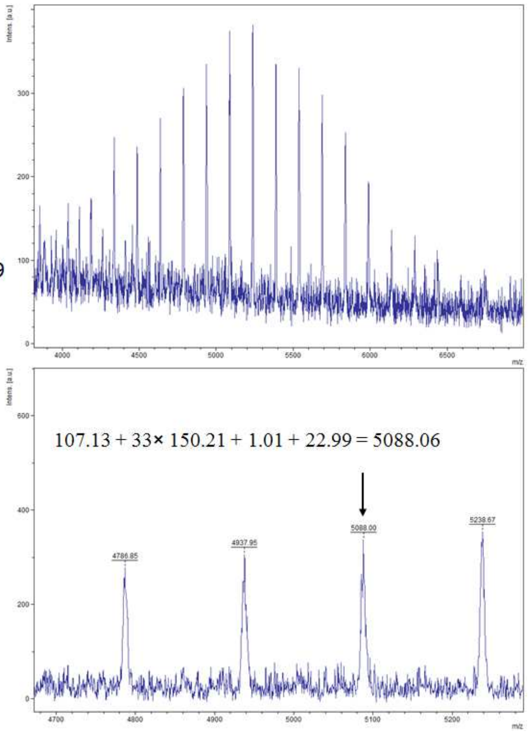


**Figure S7.** MALDI-MS analysis of **P2**

## C. Time dependent ¹H NMR spectra during the kinetics test

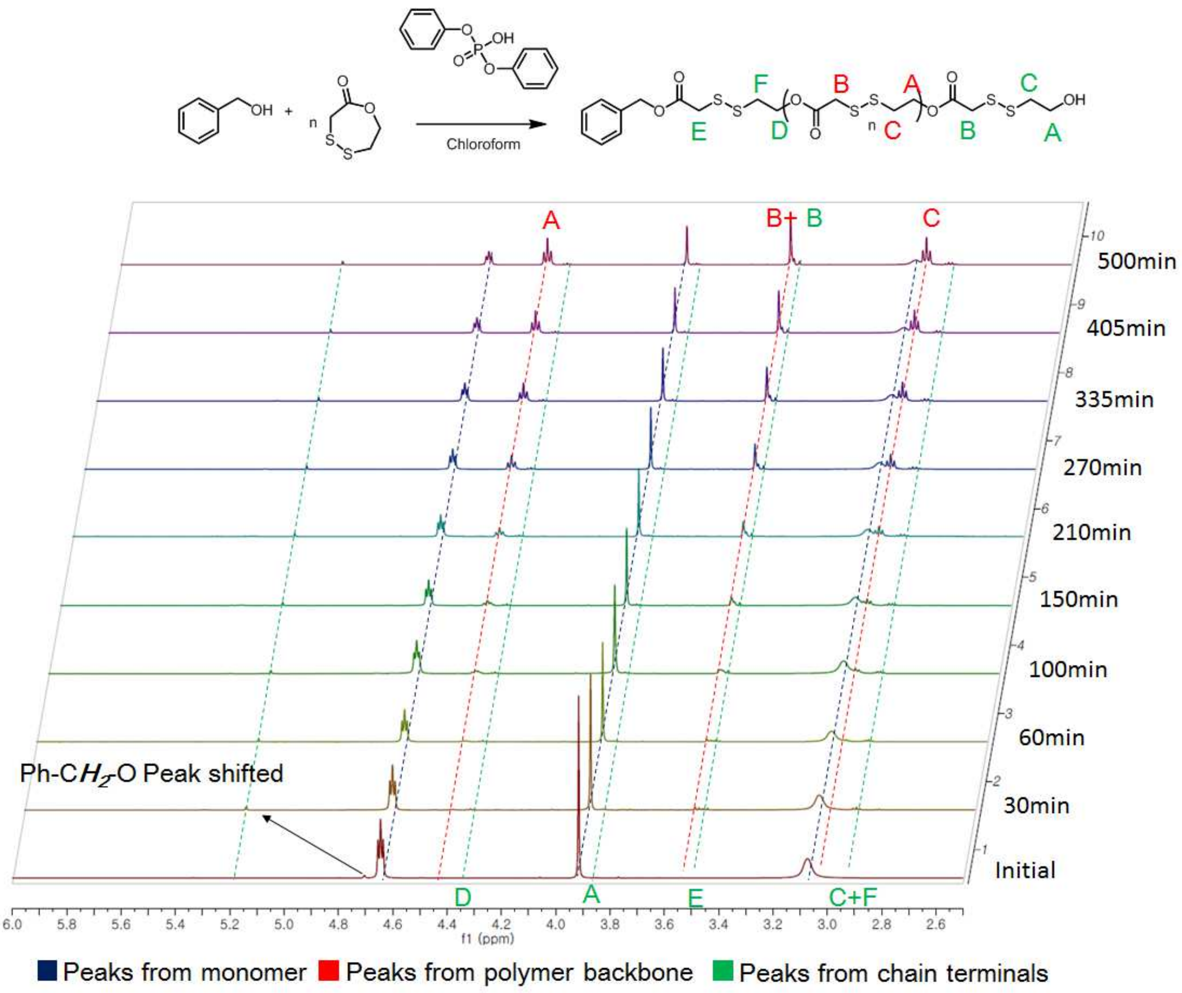


**Figure S8**. Time-dependent NMR spectra during the kinetics experiment

## D. Thermogravimetric analysis(TGA) of homopolymer P5

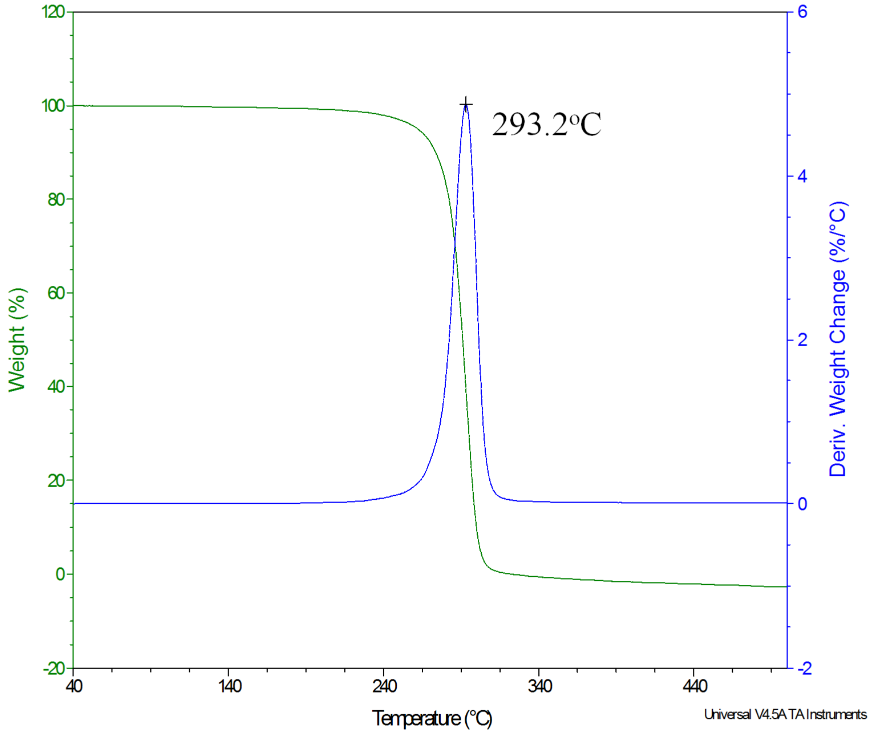


**Figure S9**. TGA analysis of **P5**

## 4. Supporting information for degradation experiments

### A. $^{1}$H NMR spectra after treating DTT

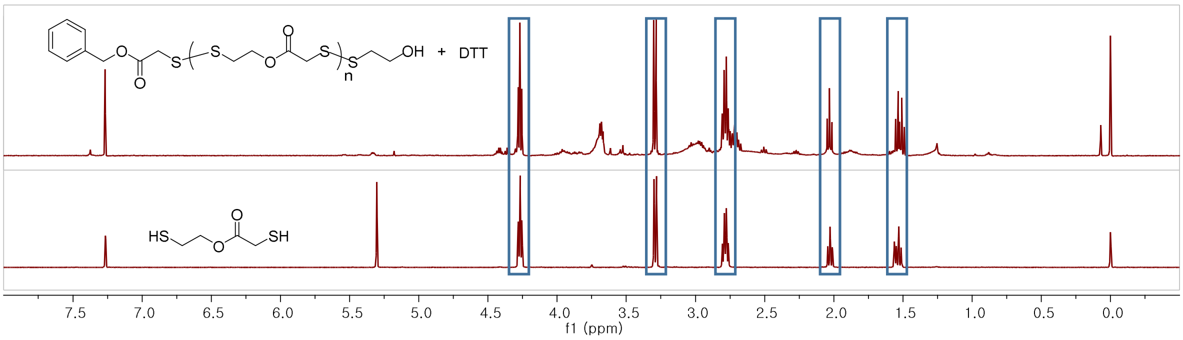


**Figure S10.** NMR spectrum of P3 after treatment of DTT

**P3** in chloroform was treated with 1 equiv. of DTT and left for 14 days in an open condition. Resulting white crude sticky solid was washed with $CDCl_3$ and $^1H$ NMR spectrum was obtained. 2-Mercaptoethyl-2-mercaptoacetate generated from thiol-disulfide exchange was observed in the spectrum.

## B. Thermal broadening of molecular weight distribution upon heating

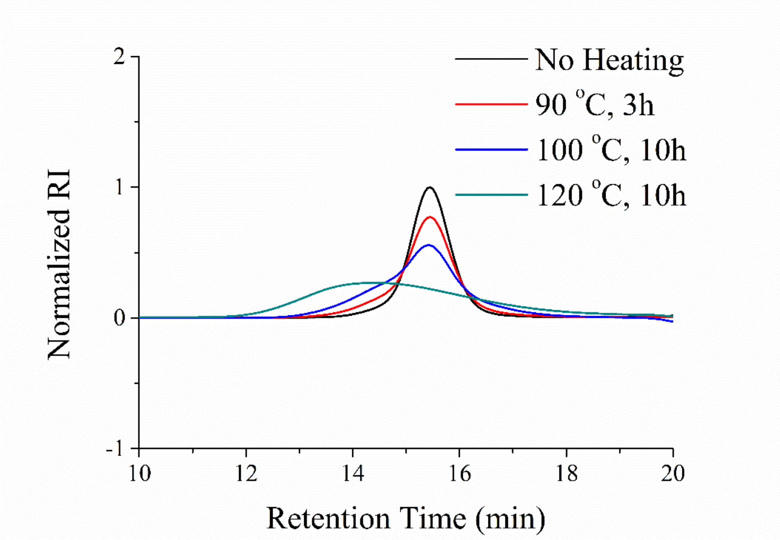


**Figure S11.** SEC chromatogram of purified poly(OTP) ($M_n$ = 6.15 kDa) before heating (black), after heating at 90 °C for 3 h (red), after heating at 100 °C for 10 h (blue) and at 120 °C for 10 h (green). The chromatogram was obtained by using THF as an eluent and polystyrene as the standard.

**Table S1.** SEC result after after heating of poly(OTP) at various temperatures.

| Temperature | Time | $M_n$ (kDa) [a] | PDI [a] |
|---|---|---|---|
| No Heating | - | 6.15 | 1.05 |
| 90 °C | 3 h | 6.18 | 1.15 |
| 100 °C | 10 h | 6.14 | 1.25 |
| 120 °C | 10 h | 6.20 | 1.85 |

[a] Determined by size exclusion chromatography (SEC), tetrahydrofuran as an eluent, calibrated with PS standards.

## C. Structural study for thermal broadening of molecular weight distribution

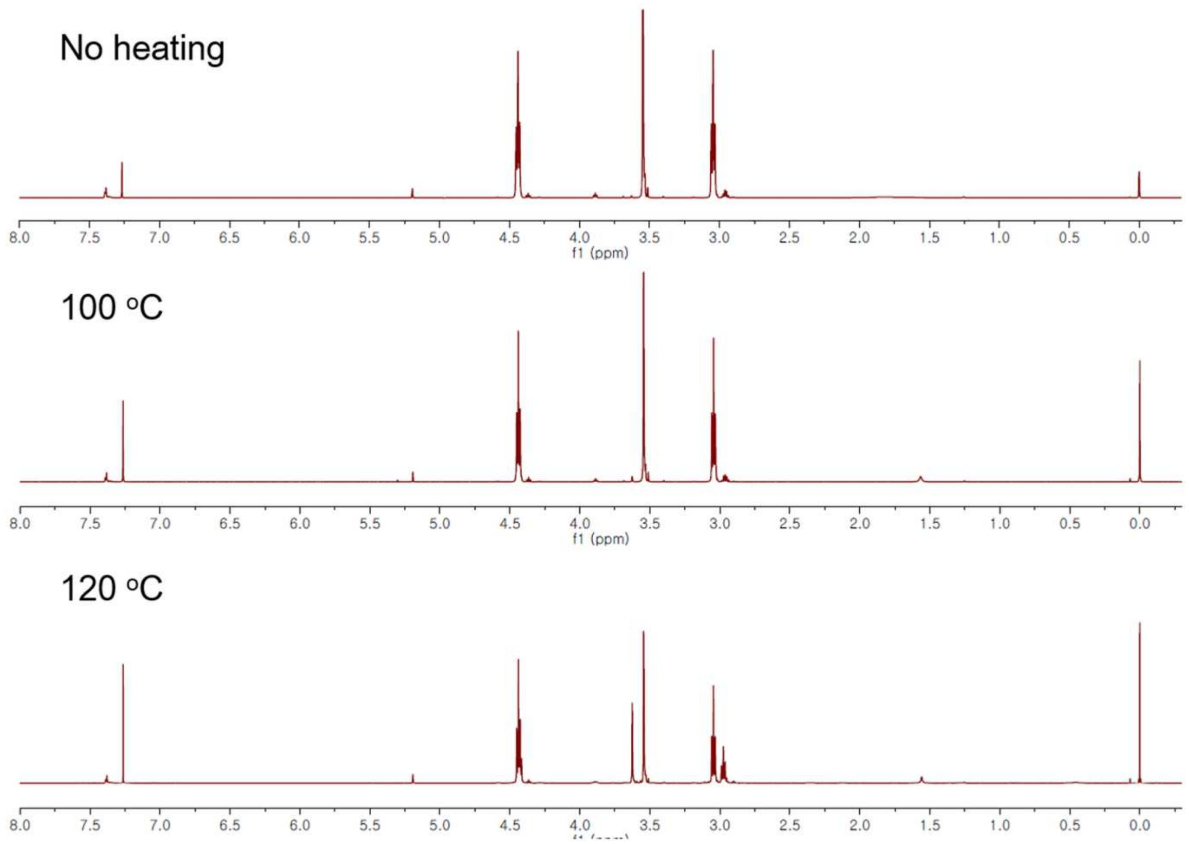


**Figure S12.** $^1$H NMR spectra of poly(OTP) ($M_n$ = 6.15 kDa) before heating (top), after heating at 100 $^o$C for 10 h (middle) and at 120 $^o$C for 10 h (bottom). (500 MHz, $CDCl_3$)

There was little change in the NMR spectrum after heating at 100 $^o$C, which indicates that only transesterification has occurred and the repeating unit of the polymer did not change. On the other hand, new peaks appeared at 2.97 ppm and 3.63 ppm after heating at 120 $^o$C, which were predicted as the product of disulfide exchange in the backbone. The possible mixture of exchange products (**H-T** (head - tail), **H-H** (head – head) and **T-T** (tail - tail) disulfides) are shown below (**Figure S13**).

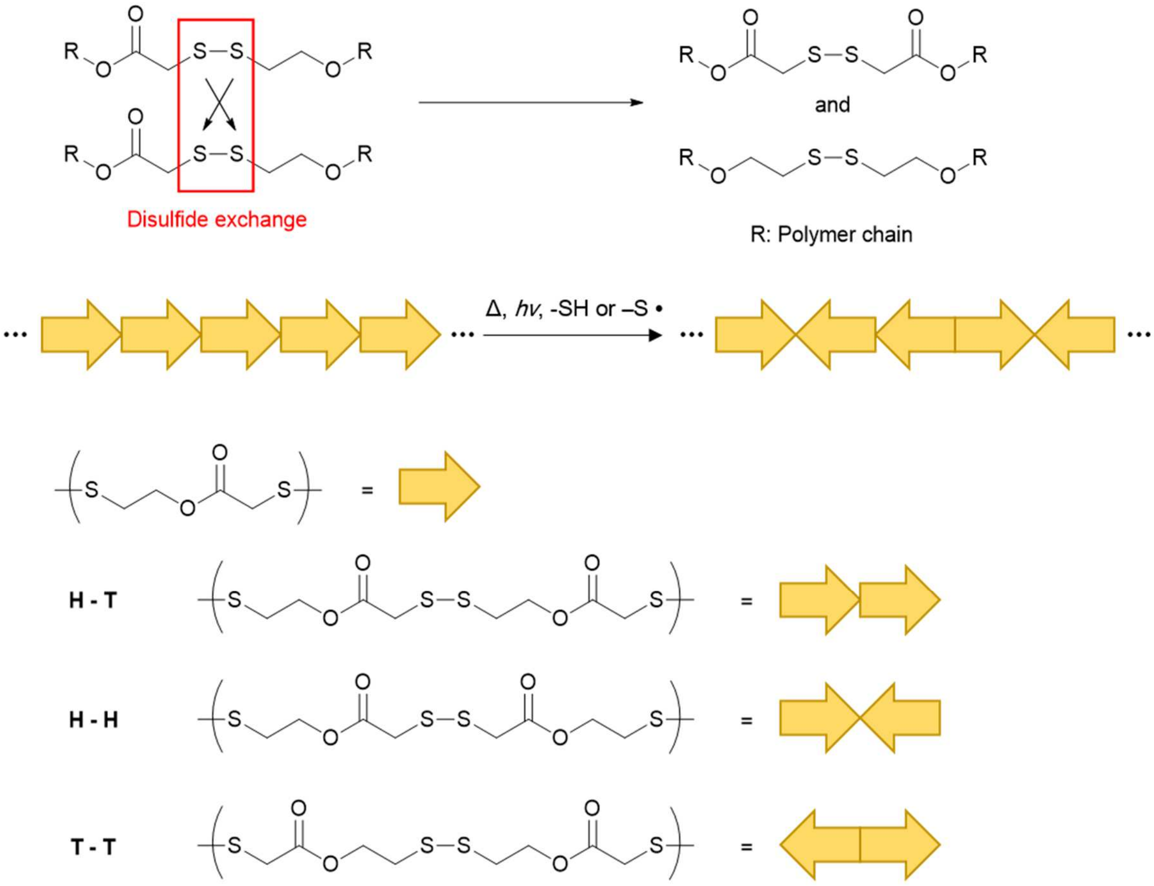


**Figure S13.** Predicted products after scrambling of disulfide bond.

If the newly generated peaks at 2.97 ppm and 3.63 ppm are surely from the disulfide exchange, the same peaks should be observable with the condensation polymerization of 2-mercaptoethyl-2-mercaptoacetate (**Figure S14**). We could observe the same peaks at 2.97 ppm and 3.63 ppm in H-NMR spectrum of the condensation product with randomly oriented repeating units (**Figure S15**).

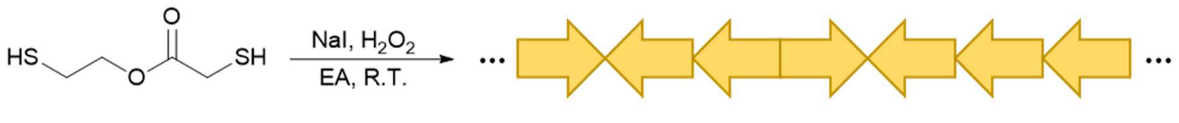


**Figure S14.** Scheme for the polycondensation of 2-mercaptoethyl-2-mercaptoacetate producing a polymer with randomly oriented repeating units. (0.3 M in ethyl acetate, 0.02 equiv. of NaI, 3 equiv. of $H_2O_2$.)

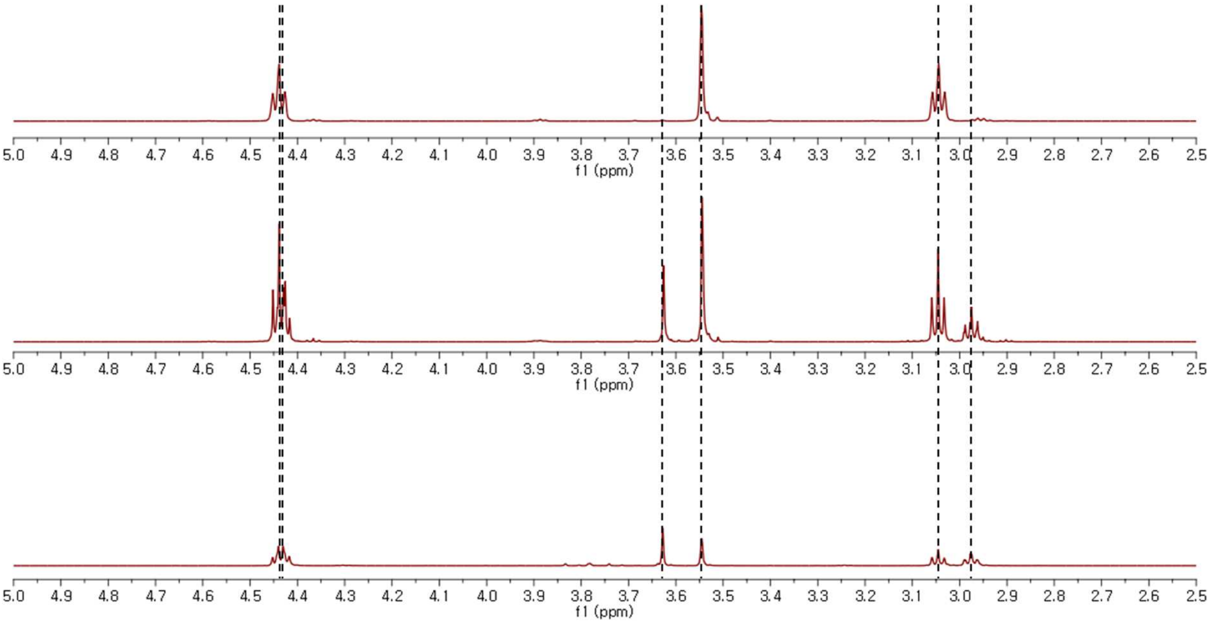


**Figure S15.** $^1H$ NMR spectra of poly(OTP) synthesized from ROP of OTPs (top), poly(OTP) after heating at 120 °C (middle) and a polycondensation product from 2-mercaptoethyl-2-mercaptoacetate (bottom).

Furthermore, model monomeric analogues of possible **H-H**, **H-T** and **T-T** backbone structures of the polymer (**Figure S16. 1, 2, 3**) were synthesized and the $^1H$ NMR spectra were compared with the condensation product of **Reaction D**. From the results, the peaks appeared after heating were confirmed as those from the products of disulfide metathesis.

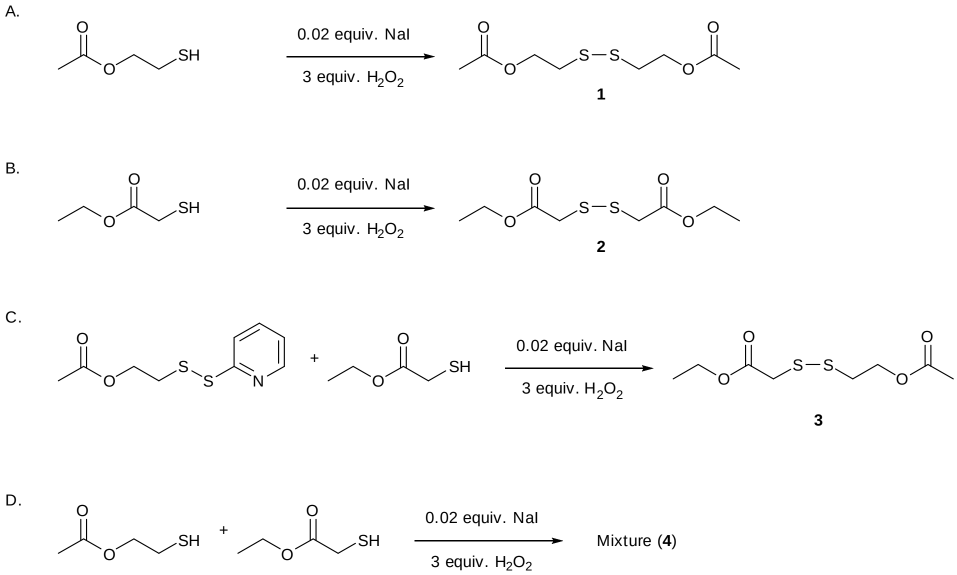


**Figure S16**. Synthesis of model monomeric analogues with possible T-T (1), H-H (2) and H-T(3) backbone structures and the mixture of the monomeric analogues (4).

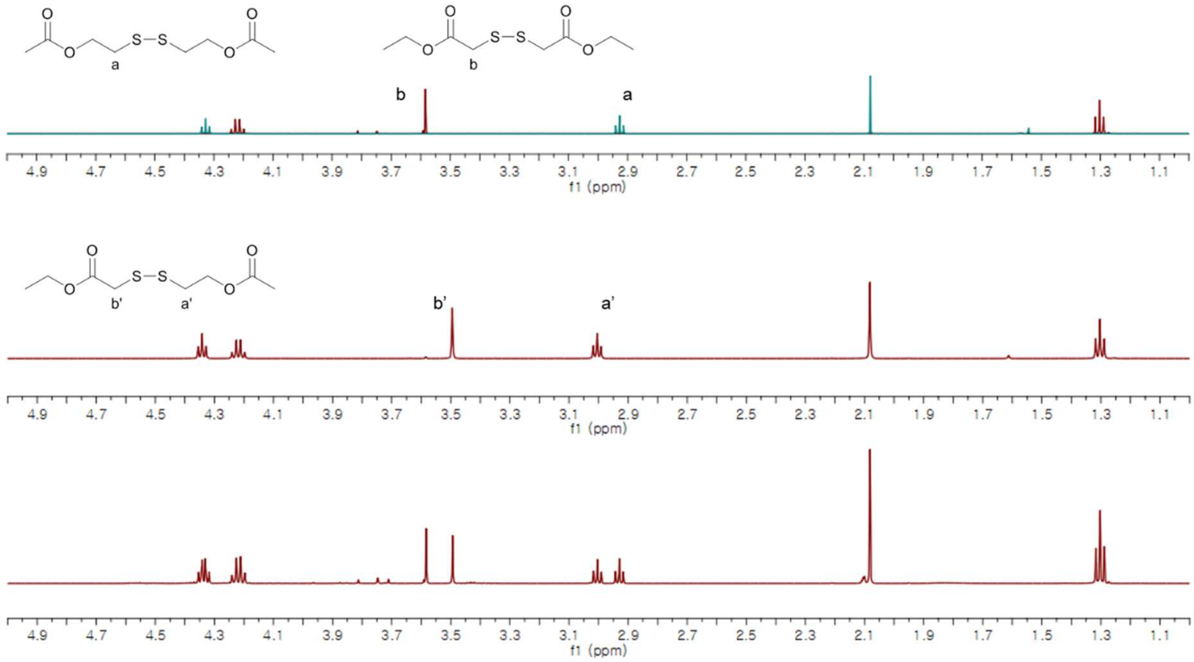


**Figure S17**. Combined $^{1}H$ NMR spectra of monomeric analogues of T-T (**1**) (top, blue) and H-H (**2**) (top, red), $^{1}H$ NMR spectrum of the H-T analogue (**3**) (middle) and $^{1}H$ NMR spectrum of the crude mixture of **reaction D** (**4**) (bottom).

## 4. NMR spectra of compounds

2-Mercaptoethyl 2-mercaptoacetate ($^1H$ NMR, 500 MHz, $CDCl_3$)

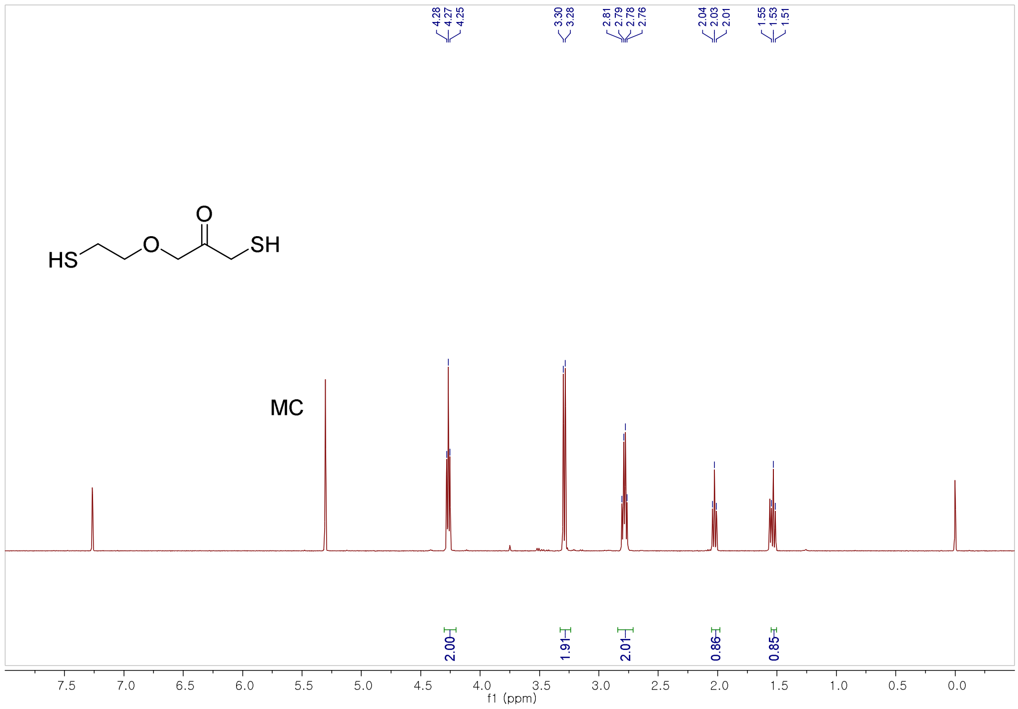


1,4,5-Oxadithiepan-2-one (OTP) ($^1H$ NMR, 500 MHz, $CDCl_3$)

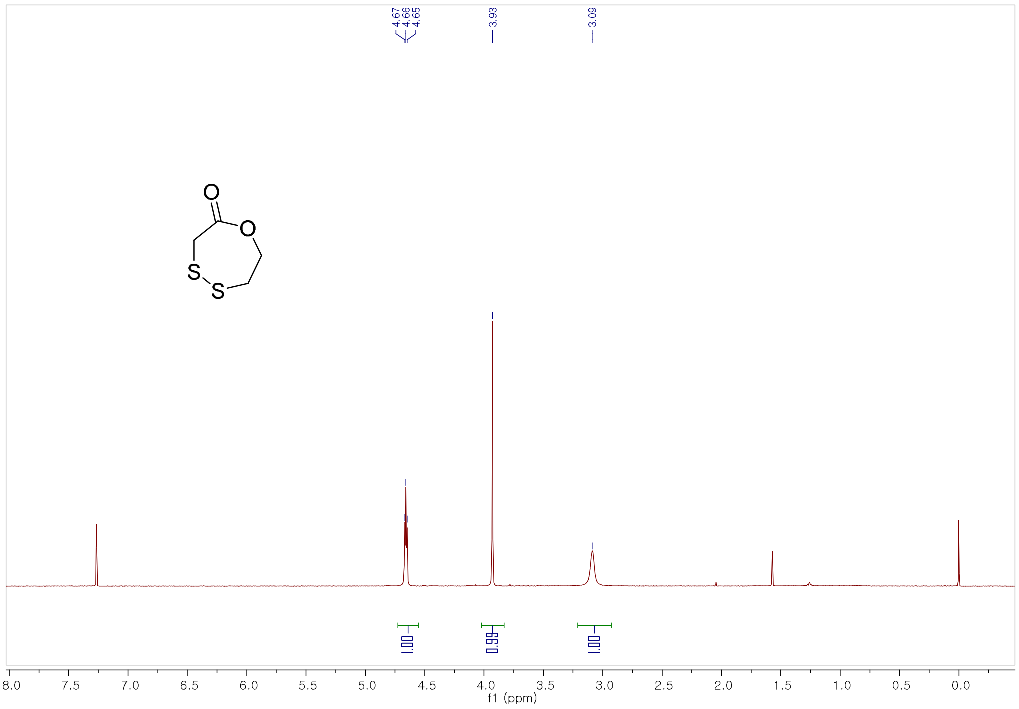

**P2** ($^1$H NMR, 500 MHz, $CDCl_3$) – Typical homopolymer initiated by BnOH

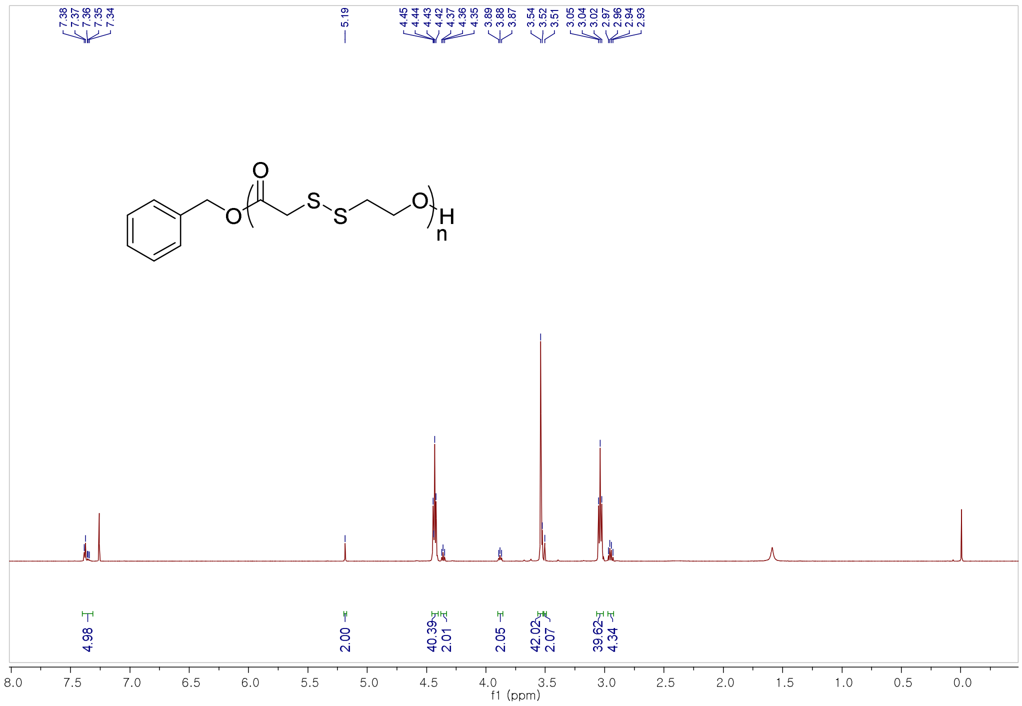


**P6** ($^1$H NMR, 500 MHz, $CDCl_3$)

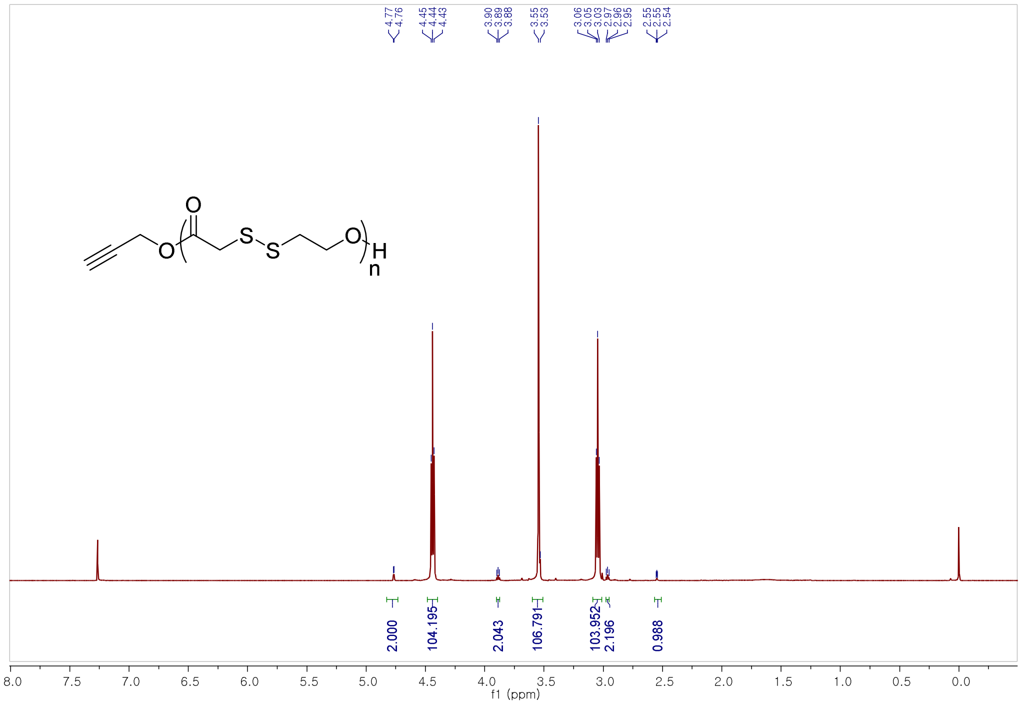

**P7** ($^1$H NMR, 500 MHz, $CDCl_3$)

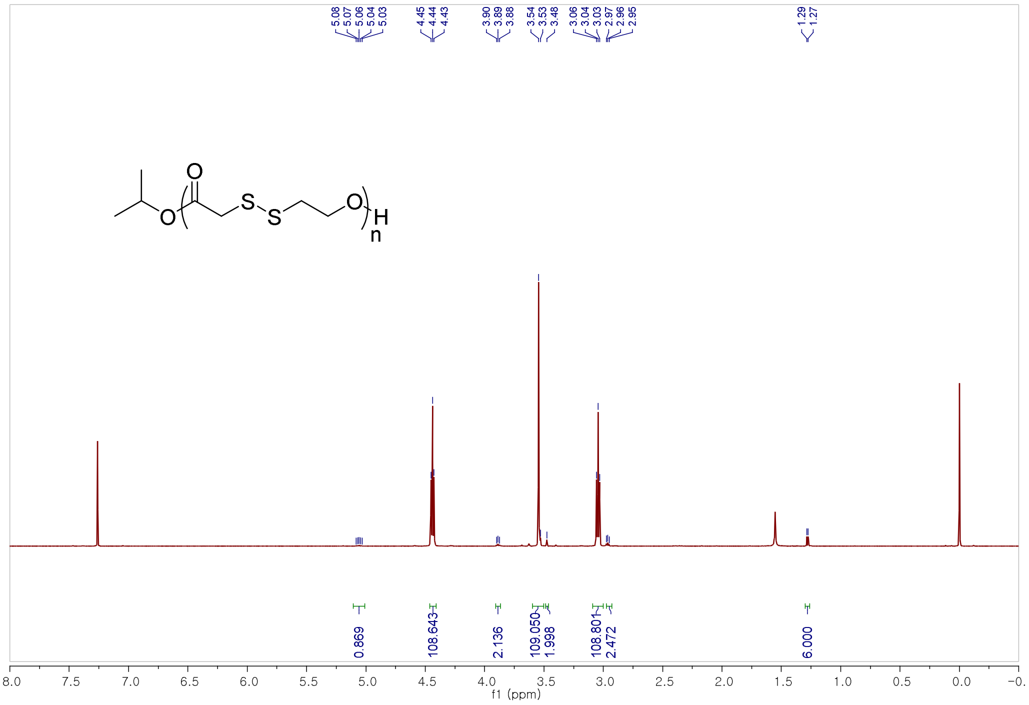

mPEO-*b*-poly(OTP) ($^1$H NMR, 500 MHz, $CDCl_3$)

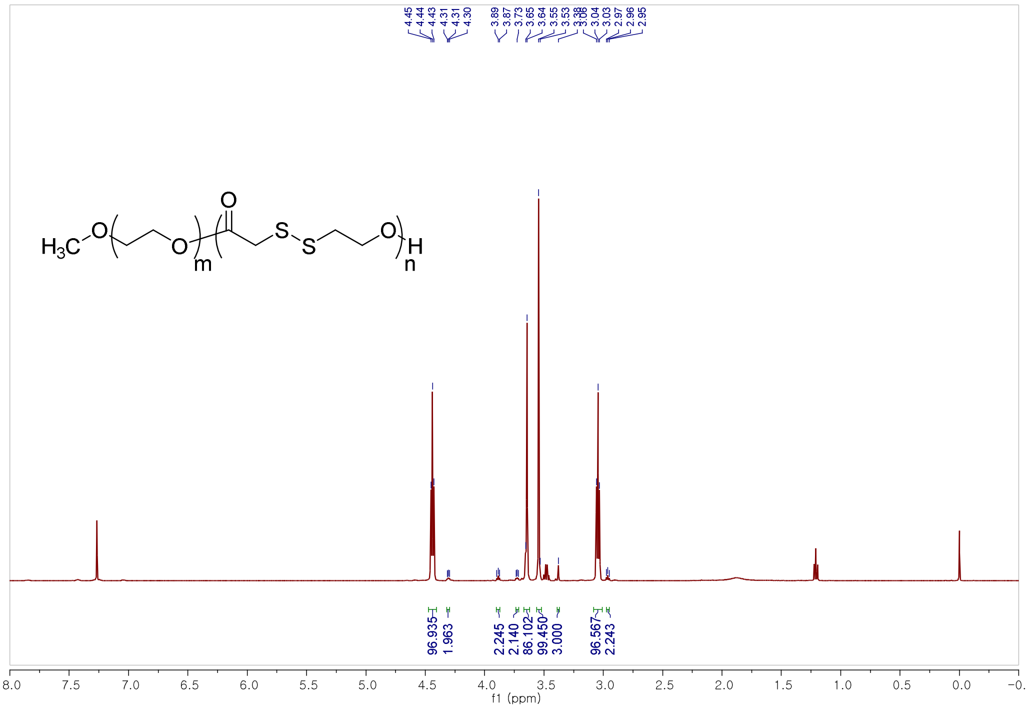

Poly(εCL)-*b*-poly(OTP) ($^1H$ NMR, 500 MHz, $CDCl_3$)

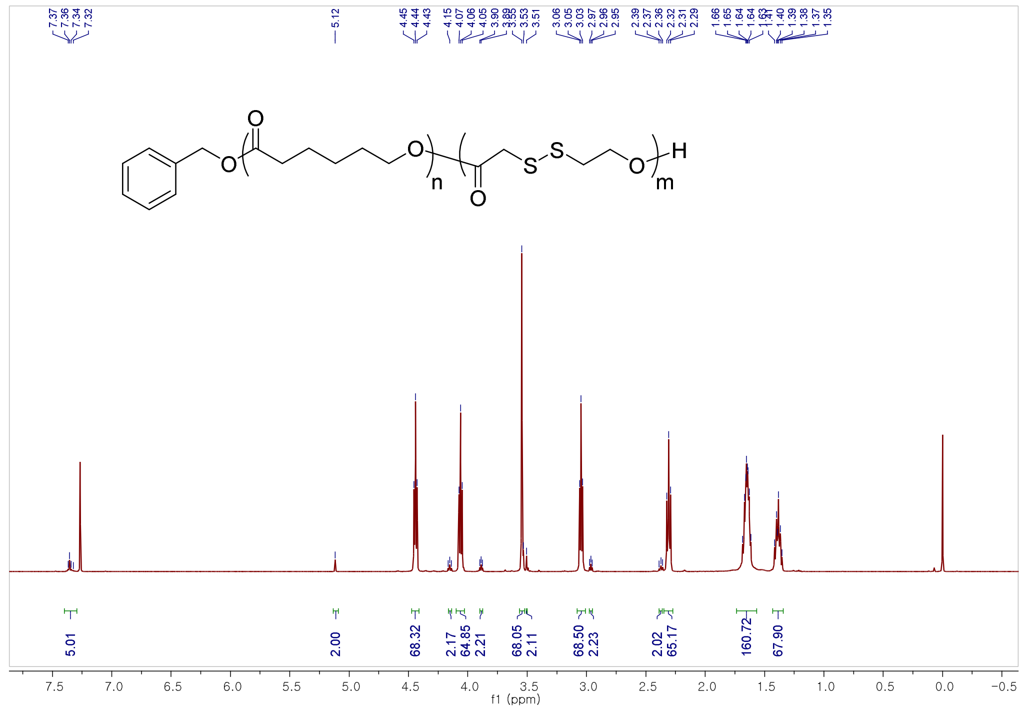

1,4,5-Oxadithiepan-2-one (OTP) ($^{13}C$ NMR, 125 MHz, $CDCl_3$)

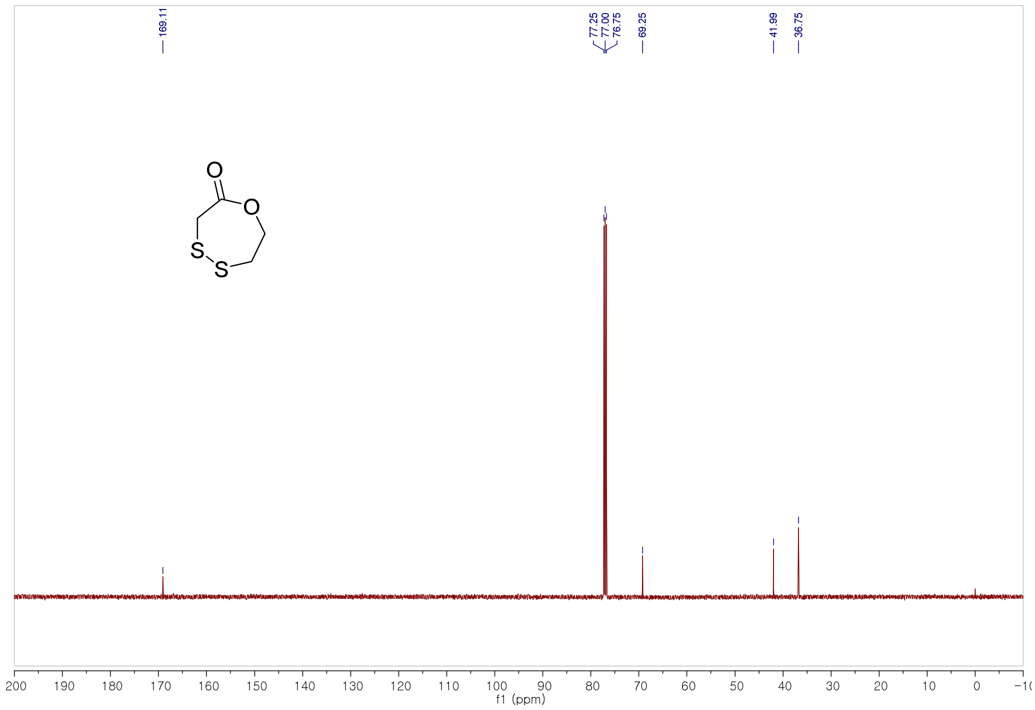

Poly(OTP) ($^{13}C$ NMR, 125 MHz, $CDCl_3$)

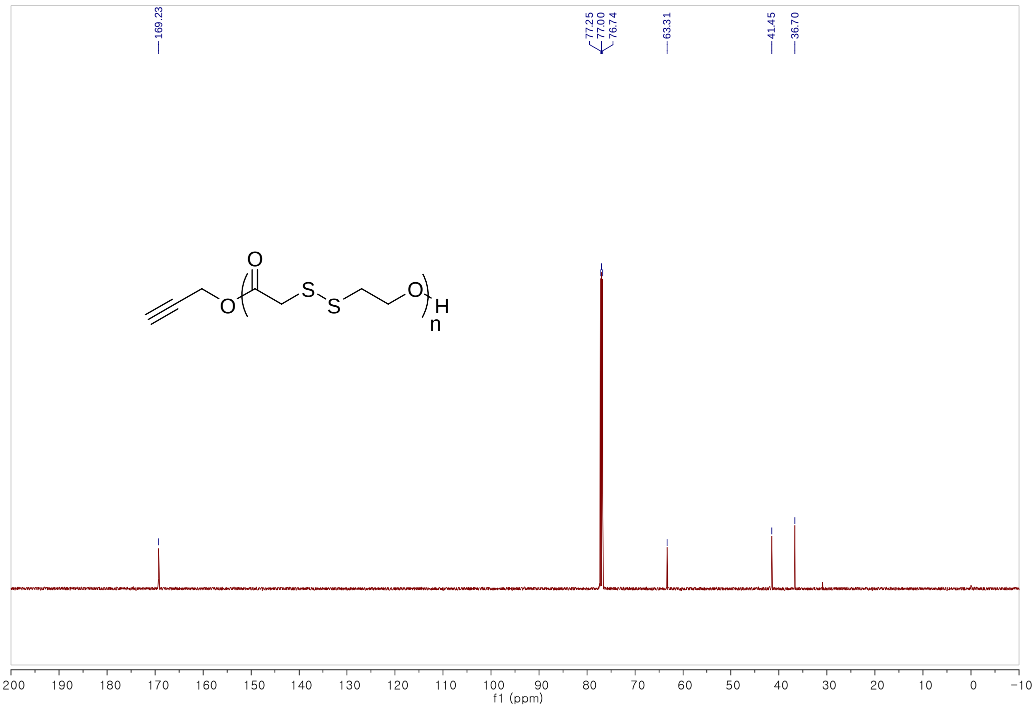


$^1H$-$^1H$ COSY spectrum of 1,4,5-oxadithiepan-2-one (OTP)

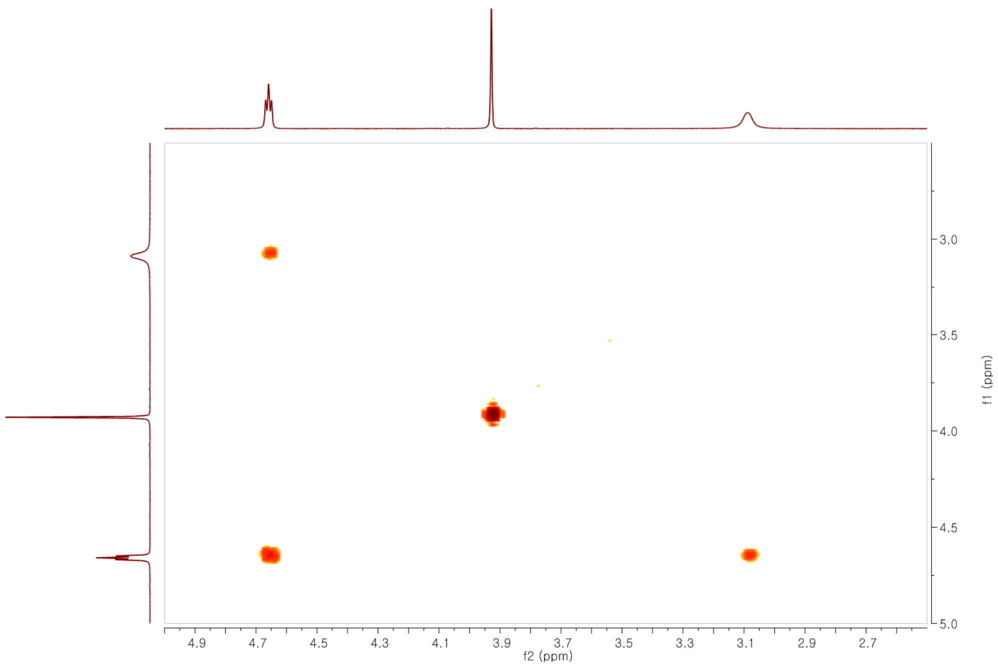

$^1H$-$^1H$ COSY spectrum of Poly(OTP)

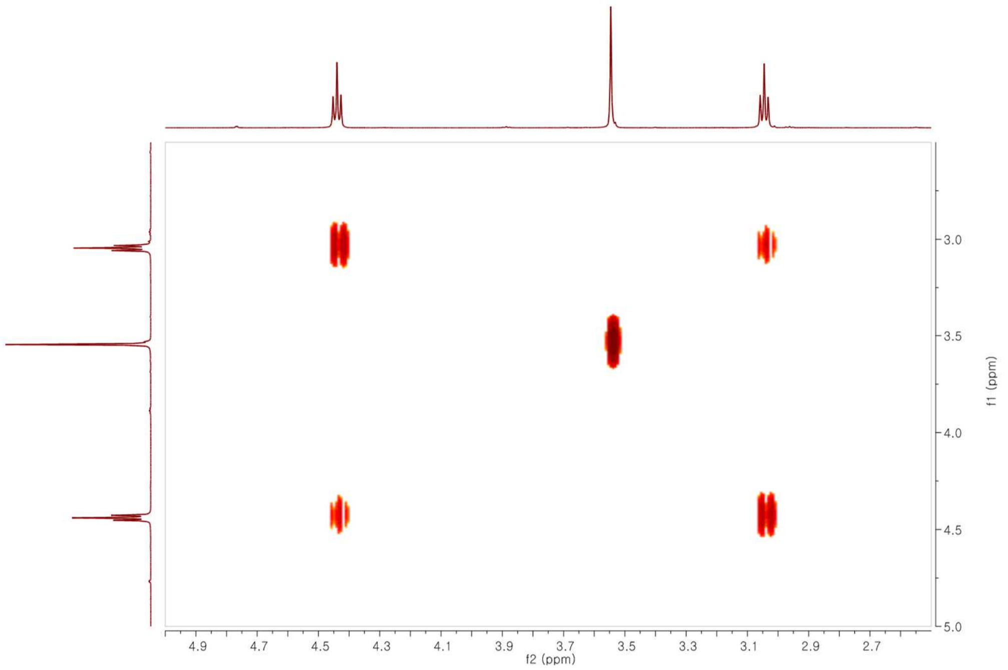

## 5. References


1. M. Kirihara, Y. Asai, S. Ogawa, T. Noguchi, A. Hatano, Y. Hirai, *Synthesis* **2007**, *21*, 3286.
